# Integrative analysis of time course metabolic data and biomarker discovery

**DOI:** 10.1186/s12859-019-3333-0

**Published:** 2020-01-09

**Authors:** Takoua Jendoubi, Timothy M. D. Ebbels

**Affiliations:** 1 Epidemiology and Biostatistics, School of Public Health, Imperial College London, Norfolk Place, London, W2 1PG UK; 2 Statistics Section, Department of Mathematics, Imperial College London, South Kensington Campus, London, SW7 2AZ UK; 3 Department of Surgery and Cancer, Imperial College London, South Kensington Campus, London, SW7 2AZ UK

**Keywords:** Metabolomics, Integrative analysis, Biomarker discovery, Pathways, Bayesian inference

## Abstract

**Background:**

Metabolomics time-course experiments provide the opportunity to understand the changes to an organism by observing the evolution of metabolic profiles in response to internal or external stimuli. Along with other omic longitudinal profiling technologies, these techniques have great potential to uncover complex relations between variations across diverse omic variables and provide unique insights into the underlying biology of the system. However, many statistical methods currently used to analyse short time-series omic data are i) prone to overfitting, ii) do not fully take into account the experimental design or iii) do not make full use of the multivariate information intrinsic to the data or iv) are unable to uncover multiple associations between different omic data. The model we propose is an attempt to i) overcome overfitting by using a weakly informative Bayesian model, ii) capture experimental design conditions through a mixed-effects model, iii) model interdependencies between variables by augmenting the mixed-effects model with a conditional auto-regressive (CAR) component and iv) identify potential associations between heterogeneous omic variables by using a horseshoe prior.

**Results:**

We assess the performance of our model on synthetic and real datasets and show that it can outperform comparable models for metabolomic longitudinal data analysis. In addition, our proposed method provides the analyst with new insights on the data as it is able to identify metabolic biomarkers related to treatment, infer perturbed pathways as a result of treatment and find significant associations with additional omic variables. We also show through simulation that our model is fairly robust against inaccuracies in metabolite assignments. On real data, we demonstrate that the number of profiled metabolites slightly affects the predictive ability of the model.

**Conclusions:**

Our single model approach to longitudinal analysis of metabolomics data provides an approach simultaneously for integrative analysis and biomarker discovery. In addition, it lends better interpretation by allowing analysis at the pathway level. An accompanying R package for the model has been developed using the probabilistic programming language Stan. The package offers user-friendly functions for simulating data, fitting the model, assessing model fit and postprocessing the results. The main aim of the R package is to offer freely accessible resources for integrative longitudinal analysis for metabolomics scientists and various visualization functions easy-to-use for applied researchers to interpret results.

## Background

Over the past few years, there has been a significant development in high-throughput omics technologies e.g. metabolomics, transcriptomics, genomics, epigenomics and proteomics along with a growing interest into joint modeling of multi-omic data [1, 2]. In metabolomics, several approaches are used to understand the response of a biological system as a function of an internal or external perturbation by monitoring “the chemical fingerprints that specific cellular processes leave behind" [3]. These chemical fingerprints are most commonly interrogated in terms of metabolite (i.e. low weight molecules) concentration, structure and transformation pathways (i.e set of chemical reactions) in order to identify biomarkers related to the studied process. Biomarker discovery consists of identifying a metabolite that has significant association patterns with a particular phenotype (disease, clinical variables, physical trait, etc) and that can be thus used as an indicator of that specific phenotype. Typical experimental platforms use analytical techniques such as nuclear magnetic resonance spectroscopy (NMR) [4] and mass spectrometry (MS) [5] to generate appropriate spectral metabolomic profiles of the studied biological system.

Metabolomic datasets are characterized by high correlation structures at different levels. For example, chromatographic correlation between two spectral peaks often results from adduct or isotopic effects whereas other correlation structures can result from peaks that represent related molecules operating within networks of chemical reactions in multiple injections. In addition, further correlation structure is present in longitudinal metabolomic studies due to repeated measurements of observations over time. Additional challenges include not only the low number of time points and samples compared to the number of metabolic variables, but also integration of a different omic data with the metabolomic data.

Metabolomic time series are often short due to experimental costs or ethical considerations. Typically, less than 10 time points are available compared to a large number of metabolic variables profiled at each time point e.g. hundreds of metabolic variables for targeted experiments and thousands for untargeted experiments. For this reason, the number of temporal patterns that can arise is limited (due to the limited number of degrees of freedom). Some temporal patterns may be repeated and thus these patterns can be induced by random processes. Models fitted to a small number of data points are prone to overfitting i.e. the model is very sensitive to small fluctuations. This can lead to a poor fitting to new data and thus a high generalisation error. It is also important to consider the number of parameters of the statistical model and make use of the simplest models in order to avoid overparametrisation. Finally, monitoring variables within and between multiple omic types can substantially enhance the understanding of the underlying biological mechanism and provide a systems biology approach as these omic variables represent entities that are often involved in related cellular processes [1, 2]. For all these reasons, metabolomics scientists need robust models which allow cautious interpretation of the data. Models are needed which integrate heterogeneous omic data and take into account both experimental conditions and biological variation.

There is a growing interest in longitudinal experiments for heterogeneous omics data and statistical models to infer biomarkers of a particular treatment or disease over time. Some approaches aim to infer influential or significant metabolites using dynamic metabolomic data under the assumption that metabolites are independent. These models include fitting smooth splines mixed effects models (SME) to time curves [6] and linear mixed effects models augmented with a variable selection [7]. However metabolomic data exhibit rich correlation structure, which is biologically relevant and should be modelled.

Seemingly unrelated regression accounts for metabolite correlation by using correlated regression errors and can be used to identify biologically significant metabolites [8, 9]. In gene expression data analysis, [10] recently proposed to use confirmatory factor analysis to capture gene-pathway relationships and a conditional autoregressive model to capture relationships between a set of pathways where a network has been constructed based on KEGG [11] pathways. The latter accounts for biological variation in the data and aids interpretation.

In the metabolomics literature, traditional frameworks for metabolomic data analysis use dimensionality reduction techniques, namely principal component analysis (PCA), partial least squares (PLS) [12] and PLS derived models (OPLS [13], O2PLS [14], OnPLS [15]) to take into account high correlations between metabolites. Extension of PLS to O2PLS and OnPLS allows for integrative analysis of heterogeneous omic data. One of the interests of PCA (PLS) derived models is to be able to visually assess whether or not there is a time effect in the data and identify metabolites that change over time by looking into time trajectories of each metabolite [16].

Extensions to PCA and PLS for longitudinal analysis include lagged PCA (PLS) and dynamic PCA (PLS) where a backshift matrix is introduced to take into account time dependency [17, 18]. Similarly, [19] used a set of piecewise orthogonal projections latent structures to describe changes between neighbouring time points. PARAFAC [20] is a multi-linear unsupervised decomposition method that can account for the multi-way variation seen in dynamic metabolomics data. Similar models such as ASCA [21] and APCA [22] also seek to account for temporal variation by combining analysis of variance (ANOVA) with PCA. Recently, dynamic probabilistic PCA (DPPCA) was proposed in [23] as a generative probabilistic model of longitudinal metabolomic data where a stochastic volatility model is used for the latent variables. The main inconvenience of these approaches is that further techniques such as variable importance scores have to be separately applied to the data in order to identify biomarkers and they do not take heterogeneous data (i.e. data from different omics techniques) into account.

In this paper, we are interested in dose-response time course experiments where additional omic variables (bacteria, genes, transcripts, etc) are monitored along with metabolites in the context of biomarker discovery. The main contribution of our work is a *single* probabilistic generative model that i) can overcome overfitting via the use of weakly informative priors ii) makes use of mixed effects models to model the experimental design iii) models metabolite interactions using pathway information through a conditional auto-regressive (CAR) component and iv) uncovers multiple associations between metabolites and other omic variables by using a horseshoe prior. An additional benefit of our approach is that it naturally yields a list of perturbed metabolic pathways.

## Results

### Model

We denote a metabolomics data set by $\boldsymbol {X} \in \mathbb {R}^{N \times T \times M}$ where *N* is the number of individuals, *T* the number of time points and *M* the number of metabolite variables (henceforth termed metabolites for simplicity). $\boldsymbol {Y} \in \mathbb {R}^{N \times T \times K}$ is an additional continuous omic data measured along with ***X*** where *K* is the number of associated omic variables. The set of *N* individuals consists of a set of cases and controls. Throughout the paper, index *i* always runs through individuals, index *t* runs through time points, index *m* runs through metabolites and index *k* runs through ***Y*** variables. Vector quantities are written in bold. Matrices are written in bold capitals. Our goal is to build a simple model that can identify biomarkers of a specific treatment over time, taking into account the multiple sources of variation in the data.

The model is built on three levels (see “Methods” section for details on full model): First, a CAR component to capture interaction between metabolites. Second, a variable selection model to uncover associations between metabolites and ***Y*** data. Third, a mixed effects component to model the experimental design. This yields the following hierarchical model:

1 $$\begin{array}{@{}rcl@{}} \boldsymbol{x}_{it}^{e} \vert \boldsymbol{\mu}_{it}, \boldsymbol{C}, \sigma \sim N \left(\boldsymbol{\mu}_{it}, \left(\boldsymbol{I}_{M}-\boldsymbol{C} \left(\boldsymbol{\phi}^{e}\right) \right)^{-1} \sigma^{2} \right)  \end{array} $$

2 $$\begin{array}{@{}rcl@{}} \mu_{itm} = \alpha_{m} + \gamma_{im} + \boldsymbol{\beta}_{m} \boldsymbol{y}_{it} + \nu_{itm}  \end{array} $$

where the mean metabolite level ***μ***_*it*_ is a function of covariates of sample *i* at time point *t*, influence of metabolite *j* on metabolite *m* is captured through coefficients *c*_*mj*_ elements of the matrix $\boldsymbol {C} \left (\boldsymbol {\phi } \right) = \sum _{p=1}^{P}\phi _{p} \mathbf {G}_{\mathbf {p}}\mathbf {A}_{\mathbf {p}}$ where *P* is the number of pathways (see “Methods” section for details). *α*_*m*_ represents treatment effect for metabolite *m*, *γ*_*im*_ represents individual perturbations for metabolite *m*, *ν*_*itm*_ represents temporal effects for metabolite *m* of individual *i* at time point *t* and ***β***_*m*_ quantifies interactions between metabolite *m* and other omic variables. The indicator *e* stands for {controls,cases} and ***ϕ*** quantifies pathway perturbation. Particularly, by specifying different dependence parameters for metabolite interactions in cases and controls, the model is able to identify perturbed pathways by comparing ***ϕ***^cases^ and ***ϕ***^controls^.

We refer to our model as “ iCARH ” model for “ integrative CAR Horseshoe ” model. The model we propose is implemented in the iCARH package and freely available from the Comprehensive R Archive Network (CRAN).

In the following sections, we perform experiments on both synthetic and real data to investigate whether our algorithm gives reasonable solutions. We first try our method on a simulated dataset in “Simulation study” section to get an understanding of the performance of our method. In “Case study” section, we test our method on a dataset comprising metabolomic and bacterial composition profiles in a drug treatment experiment. A fully worked reproducible example using the iCARH package on a publicly available dataset is available in Additional File 1. All experiments were run on a computer with an Intel i7 processor running at 2.8 GHz using 16 Gb of RAM.

### Simulation study

To get better understanding of our method and test its applicability, we first perform our approach on synthetic datasets. We will mainly focus on the ability of our model to infer pathway perturbation.

In this simulation, we first extract from the KEGG database the proportion of pathways in which a single metabolite is involved. We then use these proportions to randomly generate a binary matrix ***Z*** with dimensions *M*×*P* indicating random assignments of metabolites to pathways where *M* is the number of metabolites, *P* is the number of pathways. Each design matrix **A**_**p**_ is then equal to **z**_**p**_**zp***T* where **z**_**p**_ is the *p*th column of *Z*. We simulate pathway perturbation according to an indicator variable *ω* as follows: If *ω*=1 then pathways are perturbed hence $\phi _{p}^{\text {controls}}$ and $\phi _{p}^{\text {cases}}$ are simulated from normal distributions with different means. If *ω*=0 then $\phi _{p}^{\text {controls}}=\phi _{p}^{\text {cases}}$ and pathways are not perturbed.

The rest of the parameters is set as follows : number of bacterial variables *K*=1, number of metabolites *M*=40, number of time points *T*=7, number of samples *N*=22, number of pathways *P*=11, global parameter *τ* fixed to 1.2 (to induce a medium degree of sparsity), parameters *ν*_*itm*_,*γ*_*im*_,*μ*_*itm*_ and multi-omics time course data $ \boldsymbol {x}_{it}^{e}$ simulated according to Eqs. 1, 2, 11, and 12, respectively (See “Methods” section). Finally, we generated 10 datasets according to the simulations above in order to assess how our model infers perturbed pathways.

We set non-informative uniform priors on $\alpha _{m}, \sigma _{\gamma _{im}}, \theta _{m}, \sigma _{\mu _{m}}^{2}$. We set an informative prior on $\sigma ^{2}_{\gamma _{m}} \sim \text {inverse-gamma} \left (1, 0.1 \right)$ as we expect low variability amongst biological samples of the same group.

#### Assessing uniform and beta-like priors

**Fig. 1:**
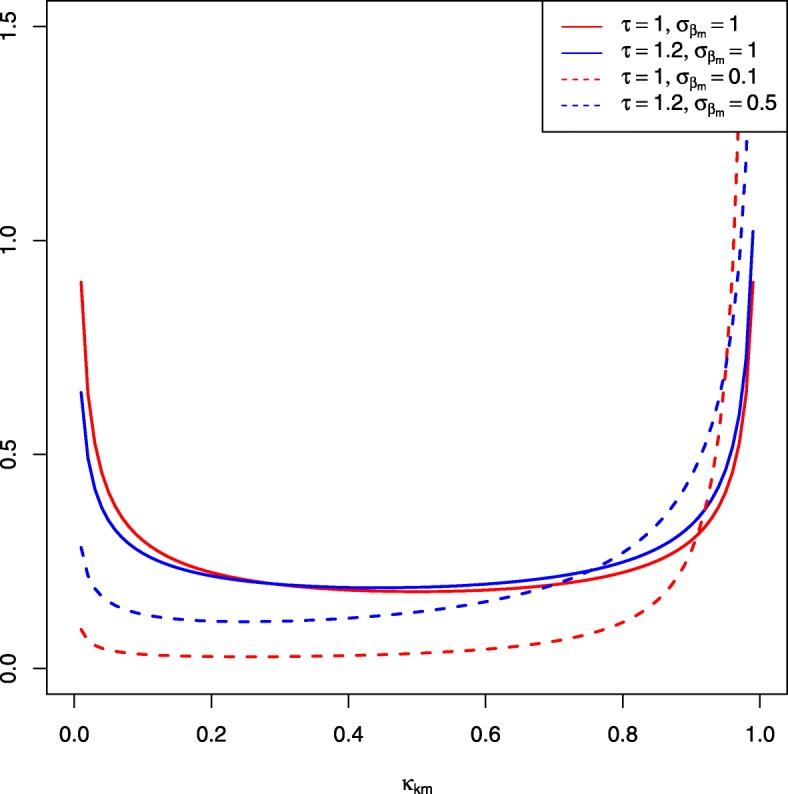
Left : Boxplots of Area Under the Curve (AUC) for pathway perturbation inference with uniform prior on ***ϕ*** compared to a beta like prior on ***ϕ*** for 10 simulated datasets. We infer perturbations based on the posterior distribution of ***ϕ***^controls^−***ϕ***^cases^. Right : Distribution of ***ϕ***^controls^−***ϕ***^cases^ under the uniform prior (white) and the beta like prior (shaded). The true perturbed pathways are printed in bold on the x axis

We first compare inference of the model under a uniform prior for $\phi ^{e}_{p}$ and the prior in Eq. 5 (See Fig. 1). Inference is done using 2000 iterations of Hamiltonian Monte Carlo sampling and 1000 warm-up iterations.

We here show, through simulation, that an informative beta like prior compares better than a non-informative uniform prior in inferring significant pathways. The left plot in Fig. 1 shows the boxplots of the 95% confidence interval of the Area Under the Curve (AUC) for pathway perturbation inference for 10 simulated datasets with uniform prior on ***ϕ*** and a beta like prior on ***ϕ***. We infer perturbations based on the posterior probability that ***ϕ***^controls^ and ***ϕ***^cases^ are different i.e. the 95% credible interval of ***ϕ***^controls^−***ϕ***^cases^ does not contain zero. The AUC values for the beta like distribution is significantly higher than the AUC values for the uniform distribution. On average pathway peturbation inference under the uniform distribution reduces to a random guess with an average AUC of 0.53. This is likely due to the lack of variance of the uniform distribution. The right plot in Fig. 1 shows the posterior distributions of ***ϕ***^controls^−***ϕ***^cases^ under the uniform prior (white) and the beta like prior (shaded) for each pathway. The true perturbed pathways are printed in bold on the x axis. In the following section, we assess pathway inference against design inaccuracies.

#### Assessing pathway inference against design inaccuracies

**Fig. 2:**
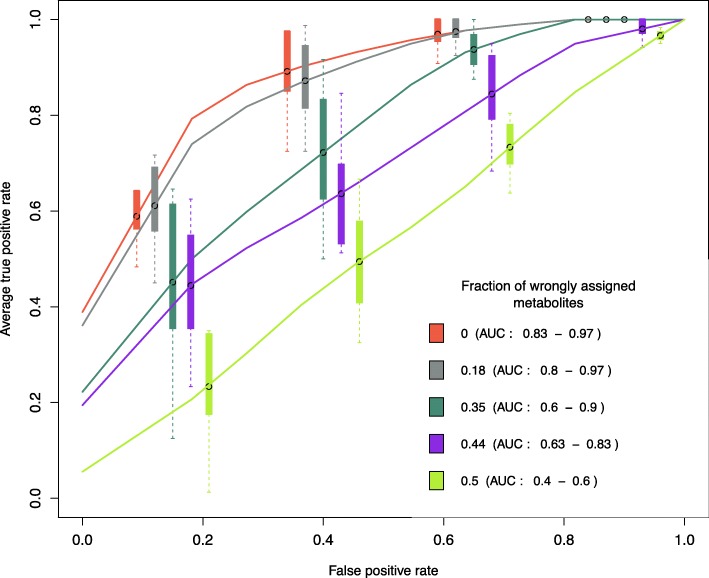
Average Receiver Operating Characteristic (ROC) curves for pathway perturbation inference across 10 datasets for different factions of “falsely” assigned metabolites. Boxplots show the 95% confidence intervals of the true positive rate

It is very common in metabolomics data to find metabolites that are correlated but not in the same KEGG pathway. In the following simulation we assess how inaccuracies in the covariance structure between metabolites and the design matrices **A**_**p**_ affect the iCARH model. We used the 10 datasets from the previous simulation and perturbed the design matrices by selecting a random fraction of metabolites in each pathway. We then randomly (falsely) assign these metabolites to no pathway, or to different pathways. We similarly run the model for 2000 iterations of Hamiltonian Monte Carlo sampling and 1000 warm-up iterations for each of the fractions {0,0.18,0.35,0.44,0.5,0.62} of perturbed metabolites. Finally, in the same fashion, we assess perturbations based on the 95% credible interval of ***ϕ***^controls^−***ϕ***^cases^. Figure 2 is a series of average Receiver Operating Characteristic (ROC) curves across 10 datasets for each of the fractions {0,0.18,0.35,0.44,0.5,0.62} of perturbed metabolites. On average, the performance of our model reduces to a random guess (AUC of 0.5) if 50% of the metabolites in each pathway is perturbed. The AUC of our model reaches 0.97 if no metabolites are perturbed and is about 0.88 if 18% of the metabolites in each pathway are perturbed.

### Case study

In this section, we test our model on an actual metabolomic data and 16S data for bacterial profiles. In this study we are interested in the influence of a diabetes drug metformin on a non-diabetic model. Metformin is the first-line medicine to treat type 2 diabetes. It has also been suggested that metformin has anti-cancer [24], cardiovascular [25] and anti-aging [26] effects. Because of their very large metabolic capacity, the gut bacteria can influence toxicity and metabolism of drugs. Here, we are particularly looking for metabolic biomarkers indicative of microbiota changes as a result of treatment.

**Fig. 3:**
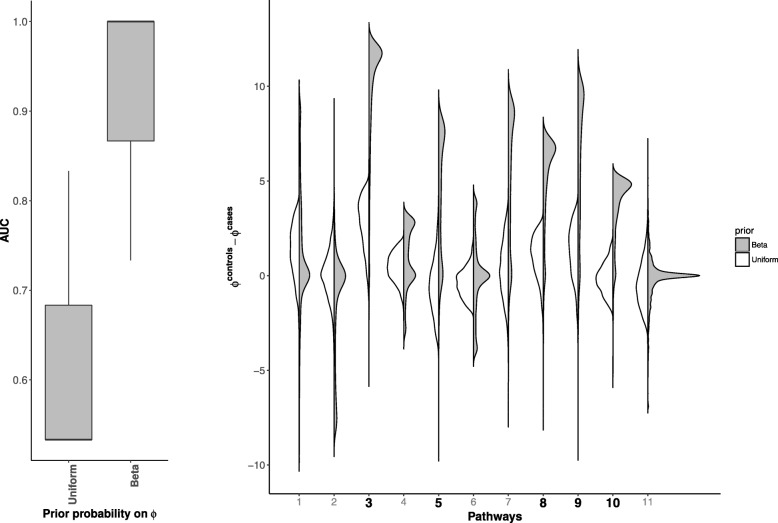
Figure shows boxplots of correlations between metabolites that are part of the same pathway (intra-pathway correlations) compared to correlations between metabolites that belong to different pathways (inter-pathway correlations) in the metformin study (See “Case study” section). Although correlations are not very close to 1. There is an empirically observed correlation signature related to whether metabolites are in the same pathway or not

The study design is as follows: metabolic profiles of 24 animals are acquired on 9 equally spaced timepoints using different mass spectrometry techniques from plasma samples. Bacterial profiles are acquired using Illumina MiSeq [27]. The study has allowed for two groups of 12 animals where the drug has been administrated to the second group (timepoints 3 to 7) allowing for an acclimatation period (timepoints 1 and 2) and a recovery period (timepoints 8 and 9). As metabolites are mapped to pathways, prior filtering and/or metabolite annotation needs to be performed beforehand. After data processing and metabolite identification, a total of 56 metabolites and 6 bacteria species are further analyzed using our model. Preliminary investigation of the data shows observable associations between correlations and inter-pathway and intra-pathway metabolites in Fig. 3 which motivates fitting the iCARH model the data. Inference is done using 2000 iterations of Hamiltonian Monte Carlo sampling and 1000 warm-up iterations.

We assess performance of our model for different values of *τ* using the Watanabe-Akaike information criterion (WAIC). Tested values of *τ* comprise 1, 1.2, 5, 10 with corresponding WAIC values of 7317.296, 7322.798, 7317.457, 7316.476 respectively. WAIC values are very similar for different values of *τ* which suggests to use the most selective model with *τ*=10 as it is the simplest i.e with the smallest number of selected variables.

#### Assessing model fit

**Fig. 4:**
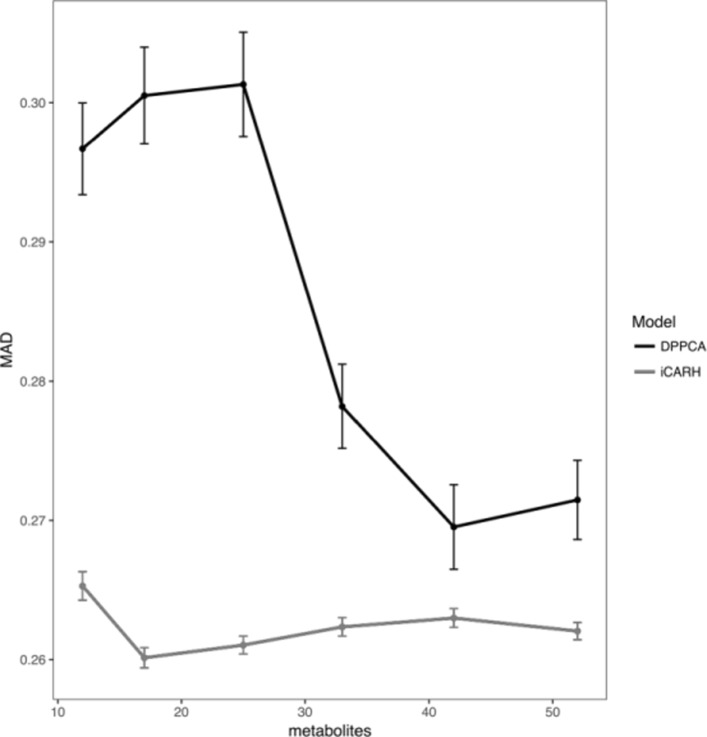
Posterior predictive checks for mean absolute deviation (MAD) compared to DPPCA for different numbers of metabolites included. Vertical bars show the 95% confidence intervals of MADs.The MADs decrease as the number of metabolites increases. Our model performs clearly better than the DPPCA model

In order to assess our model fit, we perform posterior predictive checks of our model compared to DPPCA [23]. The DPPCA model is a multivariate model using PCA, where PCA scores are modelled via a stochastic volatility model. In the Bayesian framework, posterior predictive checks consist in comparing data simulated from the posterior predictive distribution with the observed data. The mean absolute deviations (MADs) are computed between the observed covariance matrix and the covariance matrix of simulated data. The experiment was repeated for different numbers of metabolites selected randomly from the whole dataset. As metabolites are also predictors under the CAR model, the performance is expected to improve when the number of metabolites increases. The process was also replicated for inference using the DPPCA model [23]. Figure 4 shows MADs of our model and the DPPCA model. Although MADs for the DPPCA model decrease when the number of metabolites increases, it is still slightly higher than MADs for the iCARH model. Overall, our model clearly outperforms the DPPCA model.

**Fig. 5:**
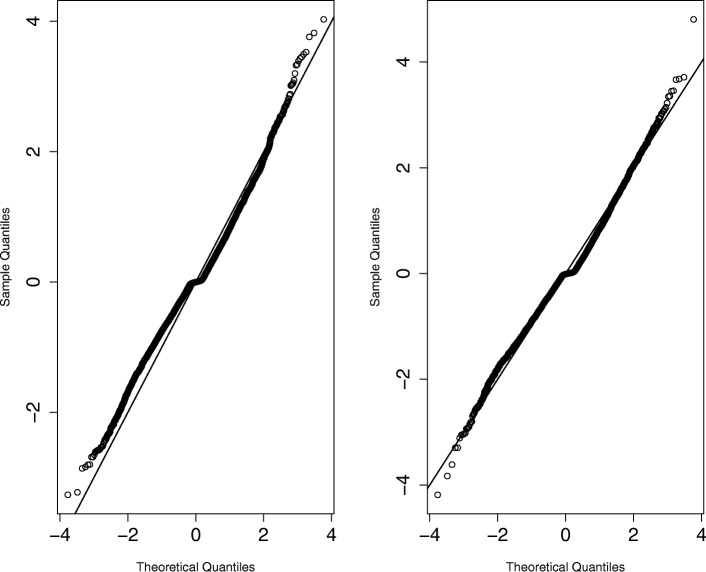
Right and left panels show model fit assessment for controls and cases for metformin data. Left : quantile-quantile normal plot of $\Psi ^{-1}_{\text {cases}} \left (\boldsymbol {x}_{it} - \boldsymbol {\mu }_{it} \right)$. Right : quantile-quantile normal plot of $\Psi ^{-1}_{\text {controls}} \left (\boldsymbol {x}_{it} - \boldsymbol {\mu }_{it} \right)$

In addition to posterior predictive checks, normality checks are another way to assess if the observed results are not mainly a product of misspecified priors. Specifically, goodness of fit was checked by using $\Psi ^{-1}_{e} \left (\boldsymbol {x}_{it} - \boldsymbol {\mu }_{it} \right) \sim N\left (0, \boldsymbol {I}_{M}\right)$ where *Ψ*_*e*_ denotes the Cholesky factor of (***I***_*M*_−**C**(***ϕ***^*e*^))^−1^*σ*^2^. Zero-mean and normality were thus checked for $\Psi ^{-1}_{e} \left (\boldsymbol {x}_{it} - \boldsymbol {\mu }_{it} \right)$ (See Fig. 5).

#### Data results

**Fig. 6:**
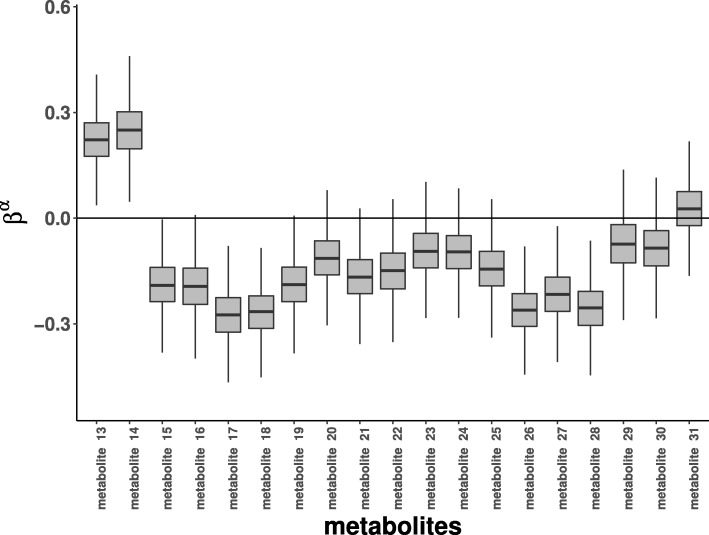
Estimates of effects of treatment on metabolite profiles are captured by $\beta ^{\alpha }_{m}$ for the metformin data. The figure depicts boxplots of 95% credible intervals of posterior means of $\beta ^{\alpha }_{m}$. Only part of the data is plotted as we are mainly interested in “metabolite 27”

In the standard model, ***α***_*m*_ represents an indicator variable for the treatment effect. The treatment variable can also be continuous as in this data example (drug measurements) and modeled by $\boldsymbol {\alpha }_{m} = \beta ^{\alpha }_{m} \boldsymbol {y}_{\text {drug}}$. The treatment effect can now be simply summarized by $\beta ^{\alpha }_{m}$. Figure 6 is a series of boxplots of 95% credible intervals of posterior means of $\beta ^{\alpha }_{m}$ for metabolites 13 to 31. We are mainly interested in “metabolite 27” as it is associated with bacteria species 2.

**Fig. 7:**
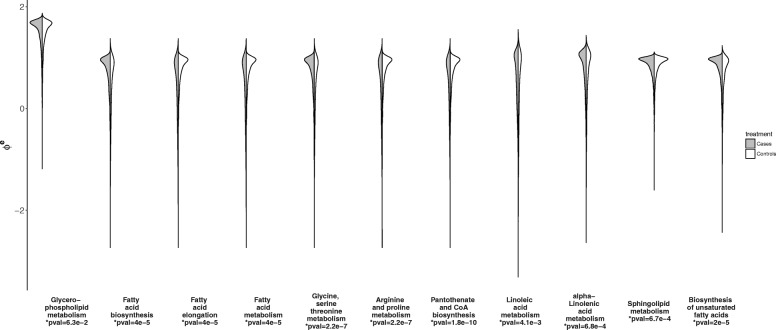
Posterior distributions of ***ϕ***^*e*^ for each pathway for each treatment group for the metformin dataset. Posterior distributions of ***ϕ***^*e*^ for some pathways are flatter for the controls than the cases which might be indicative mild pathway alterations. These pathways correspond as well to the top 11 pathways inferred by MetaboAnalyst [30]. The *p*-values are the *p*-values returned by MetaboAnalyst for each pathway

**Fig. 8:**
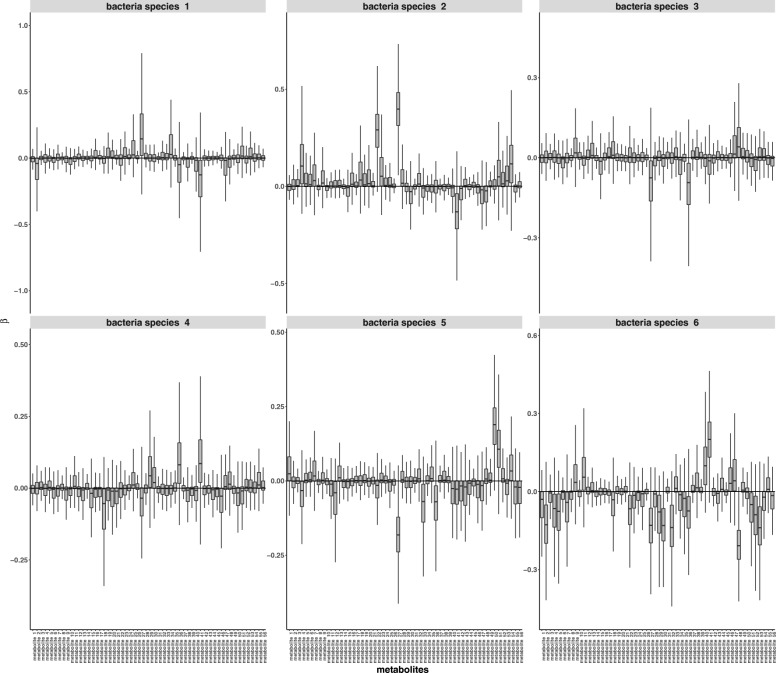
Metformin data: Estimates of effects of bacteria on metabolite profiles are captured by ***β***_*m*_. The figure depicts boxplots of 95% credible intervals of posterior means of ***β***_*m*_. Different metabolites present significant changes along with some bacterial profiles. For example, “metabolite 27” is positively associated with bacteria species 2 but negatively associated with bacteria species 5 and can be considered as indicator of changes in these bacteria species abundance

Figures 7 and 8 show posterior distributions of ***ϕ***^*e*^ for each pathway and estimates of effects of bacteria on metabolites. Results in “Simulation study” section suggest to compare the covariance structure of metabolites in the observed data with the covariance induced by the design matrices in order to have an a priori idea on the robustness of pathway inference (See Fig. 2). For a correlation threshold of 0.3, about 25% of the metabolites are misspecified in the design matrices which corresponds to an AUC around 0.8 according to Fig. 2. If we set a higher correlation threshold, a lower number of metabolites are misspecified. For example, for a correlation threshold of 0.5, only 8% of the metabolites are misspecified. This supports the use of the iCARH model for pathway perturbation inference for this data.

Estimates of effects of bacteria on metabolite profiles are captured by ***β***_*m*_. Some metabolites present significant changes along with the bacterial profiles. For example, “metabolite 27”, a hydroxy fatty acid, is associated with alterations in abundance of 4 bacteria species. Figure 7 shows that, as a result of treatment, KEGG pathways are not significantly altered. However, distributions of ***ϕ***^controls^ for “fatty acids biosynthesis” and “biosynthesis of unsaturated fatty acids” KEGG pathways are remarkably flatter than the distributions of ***ϕ***^cases^. These pathways involve the previously identified hydroxy fatty acid metabolite. Our analysis confirms previously reported studies that hydroxy fatty acids might be produced by the gut microbiome [28, 29]. On the other hand, results from MetaboAnalyst [30] give *p*-values between 6.3×10^−2^ and 1.8×10^−10^ indicating that all pathways are significant to changes of the treatment except one; glycerophospholipid metabolism. We think that this discrepancy in the results is due to the way iCARH and metaboAnalyst model pathway perturbation i.e. whilst metaboAnalyst considers that mean level changes of one or more metabolite concentrations involved in a pathway indicate perturbation of the latter pathway. iCARH considers that changes in the covariance structure of metabolites in the same pathway are indicative of pathway perturbation.

## Discussion

Identifying biomarkers in time course metabolic data and inferring significant associations with heterogeneous omic variables is extremely challenging due to the several sources of variations of the data. In addition, existing methods developed to analyse such data are very scarce and have the limitations of i) overfitting to the few available data points or ii) confounding the experimental and longitudinal variation or iii) ignoring the metabolite interactions or iv) ignoring effects of other omic variables. In this paper, the model we have developed combines several approaches to take into account the different aspects of the data namely the number of time points, the experimental variation captured by ***μ***_*it*_, interactions between metabolites captured by ***ϕ*** and interactions with additional omic variables captured by ***β***_*m*_.

Our results demonstrate that our model successfully addresses the main questions of a metabolomic study. Most importantly, our model is able to identify metabolic biomarkers related to treatment, infer perturbed pathways as a result of treatment and find significant associations with additional omic variables. We have shown that providing an informative prior on metabolic pathways and an informative prior over the parameter ***ϕ*** is a significant improvement over the DPPCA model. Particularly, our model is more robust to slight variations usually observed in short time series data thanks to the small number of covariance parameters (in the covariance matrix) needed to estimate compared to DPPCA. We have also shown through simulation that an informative beta like prior compares better than a non-informative uniform prior in inferring significant pathways. On different real data, we have investigated how the number of profiled metabolites can affect the predictive ability of the model and carried out a fully reproducible application of iCARH.

Several potential extensions arise naturally from our model. In terms of the metabolite interactions component, many research questions can arise. Alternative strategies to modeling metabolite interactions can be examined such as modeling the non-zero elements of the adjacency matrix ***C*** of each pathway as random variables. This strategy was adopted in the CAR literature by [31, 32] to take into account step changes in spatial variation. Step changes can potentially be useful to model changes in metabolites correlations as a result of treatment. Lee [33] provide an overview of different CAR models used in spatial modeling. The proposed models can be adapted to fit into the metabolomics literature.

Another potential extension concerns the source for pathway annotation and modeling. In this research paper, the KEGG pathways are used but this poses certain limitations regarding the performance of the model, due to some shortcomings in the database (missing compounds, inaccuracies in the database etc). In this sense extending the tool to support software formats and applications that enable assembly of superpathways (SBML [34], OWL [35], KEGGConverter [36] or KENEV [37]) would probably increase its performance in terms of accuracy and computational effeciency, as the adjacency matrix would be known a priori and its sparsity would be stronger.

From a practical point of view, the model has been fitted using HMC sampling but takes a large amount of time (about 1 h) mostly because of the variable selection procedure and metabolites interdependence. This could be addressed by using variational Bayes. In fact, variational Bayes inference procedures offer cost-effective inference by means of principled approximations and appealing computational time for high dimensional data. A variational bayes inference of CAR models was proposed by [38] for high dimensional data, and a variational bayes approach for variable selection was recently proposed by [39].

## Conclusion

Metabolomics longitudinal profiling techniques are imperative to understand the effect of a drug or a disease across time and can provide enhanced understanding of the underlying biology of the system. In a data integration framework, we have illustrated the use of the CAR model to incorporate metabolites interactions in the model and the horseshoe prior to identify association with heterogeneous omic variables obtained by other omic techniques. The combination of the CAR and horseshoe levels yields the “integrative CAR Horseshoe” (iCARH) model which we presented in this article. Our model is accompanied by an R package with various visualization functions easy-to-use for applied researchers.

The iCARH model has various appealing features such that it is able to identify metabolic biomarkers related to treatment, infer perturbed pathways as a result of treatment and identify potential associations between heterogeneous omic variables. Clearly, these appealing features open up further research topics.

## Methods

In this section we describe theoretical details behind the three levels of our iCARH model: Metabolite dependencies, integrative analysis with other omics data and experimental design.

### Metabolite dependencies

In any integrative biological model, it is useful to be able to interpret the model at a systems level, e.g. according to functional groups of biological molecules, rather than attempting to interpret results for individual molecules. Metabolic pathways are the most widely used groupings for this type of analysis in metabolomics, and have been widely used to interpret experimental data, usually by performing over-representation or enrichment analyses [40–44]. Since pathways are regulated in a coordinated fashion, it is natural to assume that the levels of metabolites which are members of the same pathway may be correlated. This dependence, though weak, is observable in associations between correlations and network distance [45–47], and also observable in the real data used in our study (See Fig. 3). We therefore incorporate a pathway-based correlation component into our model via a CAR approach, in a similar fashion to [10]. The extent of pathway specific correlations will vary according to the experimental system and assay, and may in some cases provide little extra information. Nonetheless, including such a pathway based component can greatly increase the interpretability of the resulting model beyond one which does not include such grouping information. In this context, some metabolite peaks in the data need to be identified in order to be mapped to pathways. As it will be later clear in the CAR model we will use, if metabolites are not identified and hence can not be mapped to pathways, no pathway-induced correlation will be assumed.

We assume that the concentration of each metabolite is linearly influenced by concentration levels of metabolites in the same pathway. Linear dependencies have been investigated in genomics in order to uncover functional modules [45]. Modeling linear associations is appealing as it captures the overall trend and also less prone to overfitting small amounts of data. Let $\boldsymbol {C} \in \mathbb {R}^{M \times M}$ be the design matrix quantifying metabolite interactions such that matrix elements *c*_*mm*_=0,*c*_*mj*_≠0 if metabolites *m* and *j* are in the same pathway and 0 otherwise. Thus, metabolite levels can be expressed as:

3 $$ {}x_{itm} \vert \boldsymbol{x}_{it,-m}, \boldsymbol{\mu}_{it}, \boldsymbol{C}, \sigma \sim N\left(\mu_{itm} + \sum_{\substack{j=1\\ j \neq m}}^{M} c_{mj} (x_{itj}-\mu_{itj}), \sigma^{2}\right)  $$

where ***x***_*it*,−*m*_ represents measurements of metabolites of sample *i* at time point *t* excluding metabolite *m*, and *μ*_*itm*_ is a function of covariates of sample *i* for metabolite *m* at time point *t* taking into account additional variation in the data (See “Integrative analysis” and “Experimental design” sections). If we define ***I***_*M*_ the *M*th order identity matrix, the joint distribution of ***x***_*it*_ can be explicitly written as [48]:

4 $$\begin{array}{@{}rcl@{}} \boldsymbol{x}_{it} \vert \boldsymbol{\mu}_{it}, \boldsymbol{C}, \sigma \sim N \left(\boldsymbol{\mu}_{it}, \left(\boldsymbol{I}_{M}-\boldsymbol{C} \right)^{-1} \sigma^{2} \right)  \end{array} $$

An important output of our modeling procedure is identification of which pathways are “on" or “off" as an effect of treatment. In the CAR literature, the design matrix ***C*** can be modeled as a scaled product of a diagonal weight matrix and an adjacency matrix. In order to infer which pathways are perturbed we construct the distance matrix based on the individual contribution of each pathway. To be precise, we define $\boldsymbol {C} \left (\boldsymbol {\phi } \right) = \sum _{p=1}^{P}\phi _{p} \mathbf {G}_{\mathbf {p}}\mathbf {A}_{\mathbf {p}}$ where *P* is the number of pathways. The distance matrices **A**_**p**_ are a zero-diagonal symmetric adjacency matrices with elements $a_{mj}^{p}$ equal to the inverse of the length of the shortest path between metabolites *m* and *j* if they are in pathway *p* and 0 otherwise. A path between two metabolites consists of the number of reactions that lead from one metabolite to the other, and the shortest path is the path that contains the smallest number of reactions. The diagonal matrices *G*_*p*_ comprise the reciprocal of the number of neighbors of each metabolite in pathway *p* i.e $ \left (g_{mm}^{p} \right)^{-1}= \sum _{j=1}^{M} (a_{mj}>0) $ so that the squared partial correlation between two metabolites $\text {cor} \left (x_{itm}, x_{itj} \vert \boldsymbol {x}_{it,-(m,j)} \right)^{2} \propto \phi _{p}^{2} g_{mm}^{p} g_{jj}^{p} $ is reduced when more metabolites from the same pathway are profiled [48]. The vector of coefficients $\boldsymbol {\phi } = \lbrace \phi _{p} \rbrace _{p=1}^{P}$ is estimated from the data. It is referred to as *spatial*-dependence parameter in the CAR literature. In the context of this work, the vector of coefficients $\boldsymbol {\phi } = \lbrace \phi _{p} \rbrace _{p=1}^{P}$ quantifies pathway contribution, for example *ϕ*_1_=0 indicates no contribution.

Under the CAR setting, we turn the reader attention that if pathway information is not available (i.e. all/some metabolites are not identified) then no pathway-induced correlation is assumed in the data and inference will be performed such that the design matrix ***C*** in Eq. 4 is a zero matrix. Hence, metabolites are assumed to be independent as the covariance matrix between metabolites is diagonal in this case.

The model needs to comply with the condition that ***I***_*M*_−**C**(***ϕ***) is positive definite. If we assume that pathways are a priori equally perturbed, *ϕ*_*p*_ must fall in the interval $\left (\frac {1}{P\xi _{p}^{1}}, \frac {1}{P\xi _{p}^{2}} \right)$ where $\xi _{p}^{1}$ and $\xi _{p}^{2}$ are the minimum and maximum eigenvalues of **G**_**p**_**A**_**p**_, respectively. In practice, strong interaction between observed metabolites of pathway *p* is reproduced in CAR models only when the scaling parameter *ϕ*_*p*_ is quite close to one of the boundaries $\frac {1}{P\xi _{p}^{1}}, \frac {1}{P\xi _{p}^{2}}$. Hence, we use a beta-type prior for *ϕ*_*p*_ that places substantial mass on large values of |*ϕ*_*p*_| [49]:

5 $$\begin{array}{@{}rcl@{}} \mathrm{p} \left(\phi_{p} \right) = \frac{1}{\mathbf{B} \left(\frac{1}{2}, \frac{1}{2} \right)} \left(\phi_{p}- \frac{1}{P\xi_{p}^{1}} \right)^{-\frac{1}{2}} \left(\frac{1}{P\xi_{p}^{2}} - \phi_{p} \right)^{-\frac{1}{2}}  \end{array} $$

where **B** is the beta function. The parameter *σ*^2^ captures variance heterogeneity in metabolite intensities and is given an inverse gamma prior **G**(*ψ*,*ψ*−1). This prior provides 2*ψ* pseudo-observations in addition to *NT* available observations. In order to build a reasonably informative prior we set *ψ*=*N*×*T*/4.

### Integrative analysis

In this section, we turn our attention to modeling the association between heterogeneous omic variables such as transcripts and metabolites. Association between omic variables involves complex processes where often only few variables are significant which motivates the use of shrinkage priors for integrative analysis and cross-omics biomarker discovery (See [50] for a review on shrinkage priors). Recently, [51] proposed the “horseshoe” prior as a prior based on a scale mixture of normals where scale parameters are modeled as the product of a global shrinkage (scale) parameter and a local shrinkage (scale) parameter. This definition allows for an additional flexibility where sparsity can be controlled at a global level for each metabolite (i.e. how many non-zero coefficients?) and a local level for each metabolite (i.e. which coefficients are non-zero?). The horseshoe prior has been widely recognized and extended by the statistical community since its introduction by [51] as it benefits from various desirable properties such as simple analytic form, easy computation and preservation of significant coefficients (no over-shrinkage) [52]. In order to model the association between heterogeneous omic variables, we extend the horseshoe prior via the following hierarchical shrinkage model by introducing an additional variable *τ* to control the overall sparsity level for all metabolites:

6 $$\begin{array}{@{}rcl@{}} \mu_{itm} = \alpha_{m} + \gamma_{im} + \boldsymbol{\beta}_{m} \boldsymbol{y}_{it} + \nu_{itm} \end{array} $$

7 $$\begin{array}{@{}rcl@{}}  \beta_{mk} \vert \lambda_{mk}, \sigma_{\beta_{m}} \sim N \left(0, \lambda_{mk}^{2} \sigma^{2}_{\beta_{m}}\right) \end{array} $$

8 $$\begin{array}{@{}rcl@{}} \lambda_{mk} \vert \tau \sim \text{St}^{+} \left(\tau,0,1 \right) \end{array} $$

where *α*_*m*_ represents the treatment effect for metabolite *m*, $ \gamma _{im} \sim N\left (0, \sigma _{\gamma _{m}}^{2} \right)$ represents individual perturbations for metabolite *m*, $\nu _{itm} \vert \nu _{i,t-1,m} \sim N\left (\theta _{m} \nu _{i,t-1,m}, \sigma _{\nu _{m}}^{2}\right)$ follows and auto-regressive process and represents temporal effects for metabolite *m* of individual *i* at time point *t*. ***β***_*m*_ is a vector of dimension *K* that quantifies interactions between metabolite *m* and other omic variables encoded in the vector ***y***_*it*_ of dimension *K*. *λ*_*mk*_ is called the local shrinkage parameter whilst $\sigma ^{2}_{\beta _{m}}$ is the global shrinkage parameter. St^+^ denotes the half Student-t distribution with *τ* degrees of freedom. For *τ*=1, this prior reduces to the horseshoe prior [51]. Intuitively, for small values of *λ*_*mk*_ the coefficient *β*_*mk*_ is very close to 0 while for relevant variables *λ*_*mk*_ will be large. In addition, $\sigma _{\beta _{m}}$ controls the overall shrinkage level i.e sparsity of the vector ***β***_*m*_ is more important for small values of $\sigma _{\beta _{m}}$.

Define $\kappa _{mk} = \frac {1}{1+ \lambda _{mk}^{2} \sigma ^{2}_{\beta _{m}}/ \tau }$ a random shrinkage coefficient such that *κ*_*km*_≈0 when *λ*_*mk*_ is large and *κ*_*km*_≈1 when *λ*_*mk*_ is small. This transformation implies the following prior distribution on *κ*_*mk*_:

9 $$ {}\mathrm{p} \left(\kappa_{mk} \vert \tau, \sigma_{\beta_{m}} \right) = \frac{1}{2 \sqrt{\pi} \mathbf{B} \left(\frac{\tau}{2}, \frac{1}{2} \right)} \frac{\sigma_{\beta_{m}}^{\tau} \kappa_{mk}^{\tau/2-1} \left(1-\kappa_{mk}\right)^{-1/2}}{\left(1- \kappa_{mk} + \kappa_{mk} \sigma_{\beta_{m}}^{2} \right)}  $$

**Fig. 9:**
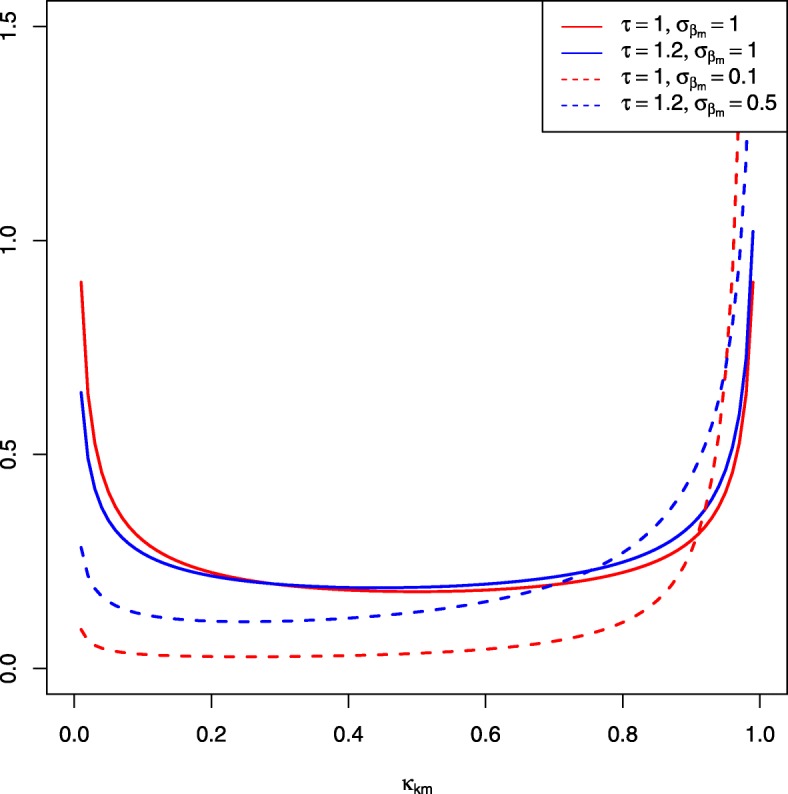
Shrinkage prior $\mathrm {p} \left (\kappa _{mk} \vert \tau, \sigma _{\beta _{m}} \right)$ on *κ*_*mk*_ for different values of $\sigma _{\beta _{m}}$ and *τ*. The prior distribution skews towards 1 if *τ* increases or $\sigma _{\beta _{m}}$ decreases (shrinkage). It skews towards 0 if *τ* decreases or $\sigma _{\beta _{m}}$ increases (no shrinkage)

This prior density is shown in Fig. 9 for different values of $\sigma _{\beta _{m}}$ and *τ*. It reduces to a Beta (*τ*/2,1/2) distribution if $\sigma _{\beta _{m}}=1$ and to a Beta (1/2,1/2) which looks like a horseshoe, if in addition *τ*=1. When *τ* increases, Beta (*τ*/2,1/2) skews towards 1 which increases the global shrinkage power. The expectation of ***β***_*m*_ given ***Y***,***κ***_*m*_,*τ*,***μ***_*tm*_ can be expressed as:

10 $$\begin{array}{@{}rcl@{}} \mathbb{E} \left(\boldsymbol{\beta}_{m} \vert \boldsymbol{Y}, \boldsymbol{\kappa}_{m}, \tau, \boldsymbol{\mu}_{tm} \right) &=& \left(\sum_{t=1}^{T} \boldsymbol{Y}_{t}^{T} \Sigma_{m}^{-1} \boldsymbol{Y}_{t} + \tau \Upsilon_{m} \right)^{-1}  \\ & & \times \sum_{t=1}^{T} \boldsymbol{Y}_{t}^{T} \Sigma_{m}^{-1} \boldsymbol{\mu}_{tm}  \end{array} $$

where $\Sigma _{m} = \left (\frac {\sigma _{\nu _{m}}^{2}}{1-\theta _{m}^{2}} + \sigma _{\gamma _{m}}^{2} \right) \boldsymbol {I}_{N}$ and *Υ*_*m*_ is a diagonal matrix of order *K* with elements 1/*κ*_*mk*_−1. Equation (10) introduces a penalty term *τΥ*_*m*_ where *Υ*_*m*_ is a metabolite specific penalty term introduced by the horseshoe prior and *τ* is a global penalty term. Precisely, *τ* captures the overall sparsity level amongst all metabolites. The expectation of ***β***_*m*_ given ***Y***,***κ***_*m*_,*τ*,***μ***_*tm*_ is very similar to the estimate of ***β***_*m*_ under ridge regression where *τΥ*_*m*_ simply reduces to *τ****I***_*N*_.

The global sparsity level can be controlled using *τ*. Increasing the global sparsity level is a desired property in omic studies, as usually we deal with a large number of omic variables where only few are important. In appendix B we discuss how *τ* can be fixed a priori.

**Fig. 10:**
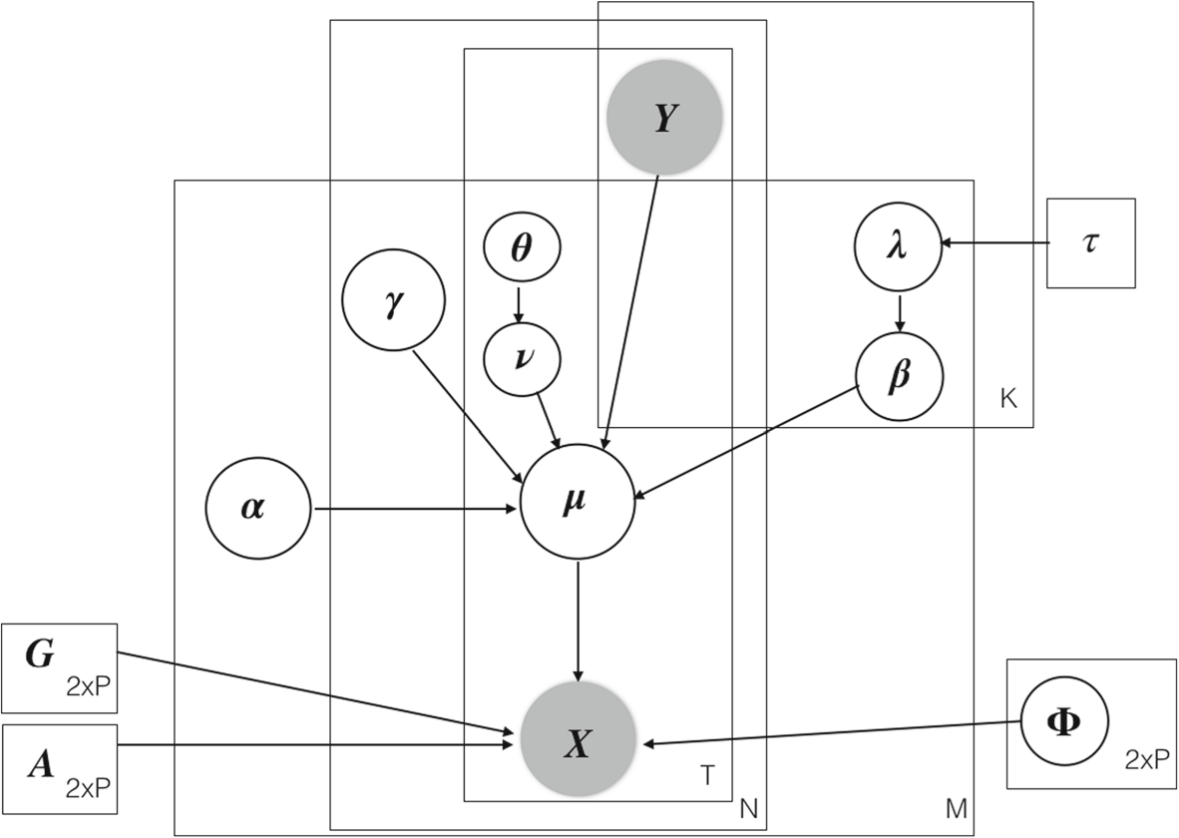
Plates diagram of the iCARH model. Fixed variables are represented by squares, random variables by circles and observations are shaded. For clarity, all variances $\sigma ^{2}, \sigma _{\beta _{m}}^{2}, \sigma _{\gamma _{m}}^{2}, \sigma _{\nu _{m}}^{2}$ are not represented in the diagram

### Experimental design

The covariance structure might change drastically as a result of treatment if the latter affects relationships between metabolites. The model can be extended to take into account the experimental design. As specified in the previous section, *α*_*m*_ captures the treatment effect for metabolite *m*, *γ*_*im*_ represents individual perturbations for metabolite *m*, $\nu _{itm} \vert \nu _{i,t-1,m} \sim N\left (\theta _{m} \nu _{i,t-1,m}, \sigma _{\nu _{m}}^{2}\right)$ represents temporal effects for metabolite *m* of individual *i* at time point *t* in Eq. 7. In addition, we allow covariance structures ***C***(***ϕ***^*e*^) to be different for the control samples and the cases where *e*∈{cases, controls} designates experimental groups. This yields the overall hierarchical model:

11 $$\begin{array}{@{}rcl@{}} \boldsymbol{x}_{it}^{e} \vert \boldsymbol{\mu}_{it}, \boldsymbol{C}, \sigma \sim N \left(\boldsymbol{\mu}_{it}, \left(\boldsymbol{I}_{M}-\mathbf{C} \left(\boldsymbol{\phi}^{e}\right) \right)^{-1} \sigma^{2} \right)  \\ \mu_{itm} = \alpha_{m} + \gamma_{im} + \boldsymbol{\beta}_{m} \boldsymbol{y}_{it} + \nu_{itm} \\ \beta_{mk} \vert \lambda_{mk}, \sigma_{\beta_{m}} \sim N \left(0, \lambda_{mk}^{2} \sigma^{2}_{\beta_{m}} \right) \\ \lambda_{mk} \vert \tau \sim \text{St}^{+} \left(\tau,0,1 \right) \\ \gamma_{im} \vert \sigma_{\gamma_{m}} \sim N\left(0, \sigma_{\gamma_{m}}^{2} \right)  \end{array} $$

12 $$\begin{array}{@{}rcl@{}} \nu_{itm} \vert \theta_{m}, \sigma_{\nu_{m}} \sim N\left(\theta_{m} \nu_{i,t-1,m}, \sigma_{\nu_{m}}^{2}\right)  \end{array} $$

A key point of this model is that by specifying different dependence parameters for metabolite interactions in cases and controls, the model is able to identify perturbed pathways by comparing ***ϕ***^cases^ and ***ϕ***^controls^.

## Appendix A: Global sparsity

When there is prior knowledge available, specifying *τ* a priori can optimize the inference and additionally, provide a more informative prior on *λ*_*mk*_. If we fix $\mathrm {p}\left (\sigma _{\beta _{m}}^{2}\right) \propto 1/\sigma _{\beta _{m}}^{2}$, integrating over $\sigma _{\beta _{m}}$ gives the expected value of *κ*_*mk*_ as :

$${\begin{aligned} \mathbb{E} \left(\kappa_{km} \vert \tau \right) = \frac{\Gamma \left(1/2\right)^{-1}}{2 \sqrt{\pi} \Gamma \left(\tau/2\right)} \mathbf{G}_{3,3}^{2,3} \left(\begin{array}{ll} 1, \tau/2, 0 \\ \tau/2, \tau/2-1/2, 0 \end{array} \, \middle\vert \, 1-\sigma_{\beta_{m}}^{2} \right)  \end{aligned}} $$

where $\mathbf {G}_{\cdot,\cdot }^{\cdot,\cdot } $ is Meijer’s G-function [53]. The equation above can be used to fix *τ* a priori by defining the expected proportion of shrunk coefficients. In practice, different values of *τ* are plugged into the equation above to get the desired proportion of shrunk coefficients. However, many definite integrals can be obtained using the tables of Meijer functions in [54] for special values of parameters.

**Table 1:** iCARH model summary

Parameters of interest	
***ϕ***^*e*^,*e*∈{cases, controls}	quantifies metabolite interactions,
	(***ϕ***^*cases*^−***ϕ***^*controls*^) quantifies
	pathway perturbation
***β***_*m*_	quantifies association between
	metabolite *m* and other omic
	variables from different omics techniques
*α*_*m*_	quantifies treatment effect for metabolite *m*
Other inferred parameters	
*σ* ^2^	metabolite variance
*γ*_*im*_	represents individual
	perturbations for metabolite *m*
*ν*_*itm*_	represents temporal
	effects for metabolite *m*
	of individual *i* at time point *t*
*θ*_*m*_	temporal dependence for metabolite *m*
*λ*_*mk*_	local shrinkage parameter
$\sigma ^{2}_{\beta _{m}}$	global shrinkage parameter
$\sigma ^{2}_{\gamma _{m}}$	variance of individual perturbations
$\sigma ^{2}_{\nu _{m}}$	temporal variance
User specified parameters	
***G***,***A***	between metabolite adjacency matrix
*τ*	overall sparsity level
	amongst all metabolites

### Appendix B: Model summary

Table 1 depicts a summary of model parameters specifying parameters of interest, other inferred parameters and user specified parameters. Figure 10 shows the plates diagram of the iCARH model where fixed variables are represented by squares, random variables by circles and observations are shaded. For clarity, all variances $\sigma ^{2}, \sigma _{\beta _{m}}^{2}, \sigma _{\gamma _{m}}^{2}, \sigma _{\nu _{m}}^{2}$ are not represented in the diagram.

The choice of the gamma distribution for $ \sigma _{\beta _{m}}^{2}, \sigma _{\gamma _{m}}^{2}, \sigma _{\nu _{m}}^{2}$ follows the same principle used in “Model” section for *σ*^2^. For each variance parameter, the gamma prior provides half pseudo-observations in addition to the available observations e.g $\sigma _{\nu _{m}}^{2}$ has a ***G***(*T*/4,*T*/4−1) prior such that it provides *T*/2 pseudo-observations in addition to *T* observations so that the prior is reasonably informative.

## Supplementary information

**Additional file 1** Worked example. We illustrate a fully reproducible application of the iCARH package to a publicly available dataset from [55].

## Data Availability

The proposed method has been implemented in the R package iCARH that is available from CRAN. A fully worked example with publicly available data is in Additional file 1.

## Abbreviations

CAR: Conditional auto-regressive model
DPPCA: Dynamic probabilistic PCA
iCARH: Integrative conditional auto-regressive horseshoe model
PCA: Principal component analysis
PLS: Partial least squares
O2PLS: Two-way orthogonal partial least squares
