# Supplementary material for: Integrative analysis of time course metabolic data and biomarker discovery
Source: BMC Bioinformatics. 2020 Jan 9;21:11. doi: 10.1186/s12859-019-3333-0 (PMC6953149; doi:10.1186/s12859-019-3333-0)
Supplement: Supplementary file 1 — Additional file 1 Worked example. We illustrate a fully reproducible application of the iCARH package to a publicly available dataset from [55]. [file 12859_2019_3333_MOESM1_ESM.pdf]

1 Worked example

In this section we illustrate a fully reproducible application of the `iCARH` package to a publicly available dataset from Brunk et al. (2016). Brunk et al. (2016) carried out a three step framework to capture experimental design, examine metabolic correlations and integrate multi-omics data respectively with each step achieved independently from the others. The dataset comprises metabolomic and proteomic time series from eight pathway engineered strains of E.coli and a wild-type E.coli DH1. Our analysis includes the eight strains where the mevalonate pathway has been adapted to produce isopentenol (I1, I2, I3), limonene (L1, L2, L3) and bisabolene (B1, B2). Each of the isopentenol-producing strains and limonene-producing strains comprise three subtypes of production performance $1, 2, 3$ indicating low, medium and high production respectively with 1 being the unoptimized strain. The bisabolene-producing strain comprises two subtypes of performance 1 and 2 with 1 being the unoptimized strain as well. Time measurements were taken at 7 timepoints starting from 0h (induction) to 36h.

The dataset is subject to incompleteness and several metabolites have missing measurements. Before starting the analysis, extracellular measurements and metabolites with more than 25% of missing values where removed. About half of the remaining metabolites have 15% of missing values. Additionally, the metabolomics and proteomics data were both centered with respect to the control group. In our analysis, the control group consists in the wild-type strain and each of unoptimized strains from the isopentenol-producing strains, limonene-producing and bisabolene-producing strains whereas the cases group contains all the optimized strains. The curated dataset can be loaded from "multiomics_ Ecoli.rda" file using

```
R> load(file="multiomics_Ecoli.rda", verbose = T)

Loading objects:
  X
  Y
  Z
  keggids
```

where `X` is the metabolomics data array with dimensions 7 timepoints $\times$ 9 strains $\times$ 29 metabolites. `Y` is the proteomics data array with dimensions 7 timepoints $\times$ 9 strains $\times$ 79 proteins. `Z` contains group information and have dimensions 7 timepoints $\times$ 9 strains and `keggids` are corresponding KEGG identifiers for metabolite names.

Model fitting and assessing model fit

In order to include pathway information into the model, metabolite names used in the dataset need to be mapped to KEGG metabolite identifiers [1]. In practice, this mapping

[1] Note that if KEGG pathway information is missing, `iCARH` will assume independence between metabolites.

was done using the MetaboAnalyst online tool (Chong et al., 2018) and corresponding KEGG identifiers stored in `keggids` variable. It should be noted that `iCARH` supports unknown KEGG identifiers entexttd by `Unk<x>` where `<x>` is an integer so that no pathway information will be considered for the corresponding metabolites. The package also supports multiple KEGG identifiers per metabolite which is not an infrequent case in metabolomics, usually occuring due to uncertainty in the exact identification (e.g. uncertainty between D- and L-alanine). To obtain the pathway adjacency matrices for the metabolomics data:

```
R> library(iCARH)
R> database = iCARH.getPathwaysMat(keggids, "sce")
```

As some pathways (such as "Biosynthesis of antibiotics") are biologically irrelevant, we reduce the list of pathways as follows:

```
R> keep = c(1,2,5,7,8,9,13,14,15)
R> pathways = database[keep]
```

and fit the model:

```
R> rstan::rstan_options(auto_write = TRUE)
R> options(mc.cores = 2)
R> set.seed(654658)
R> fit = iCARH.model(X, Y, Z, pathways,
+                    control = list(adapt_delta = 0.999,
                     max_treedepth = 15, stepsize = 0.01),
+                    iter = 3000, warmup = 1000, chains = 2)
```

We fit the model using the default value for τ, however this can be set freely by user. The `pathways` variable contains pathway adjacency matrices but in practice gene ontology classes or other molecular signatures instead of metabolic networks can be used. The model takes about 10 hours to fit using NUTS sampler on our Intel i7 2.8 GHz processor. A summary of the model parameters (by means of illustration, the $\boldsymbol{\alpha}$ parameter here) can be obtained by running the `iCARH.params` function as below. Note that the function also checks for convergence and throws a warning if convergence diagnostics fail.

```
R> iCARH.params(fit, pars="alpha")
```

```
Rhat values do not indicate any problems.
Response:  Binary.
Data: 7  time points,  9  observations,  9 pathways.
 X has  29  variables, Y has  79  variables.
MCMC samples: 2  chains,  3000  iterations each with  1000  warmup samples.

Treatment Effect (alpha):
                              Estimate MC.Error Est.Error  2.5% 97.5%
Glucose.g.L                      -0.52   0.0105      0.51 -1.54 0.449
Pyruvate.g.L                      0.42   0.0099      0.50 -0.61 1.370
Succinate.g.L                     0.12   0.0144      0.58 -1.04 1.221
Lactate.g.L                      -0.29   0.0130      0.55 -1.37 0.772
Formate.g.L                      -0.41   0.0111      0.53 -1.48 0.578
Acetate.g.L                      -0.80   0.0121      0.56 -1.90 0.323
Intracellular.Mevalonate..uM.     0.20   0.0189      0.51 -0.88 1.128
...
                              Eff.Sample Rhat
Glucose.g.L                         2393    1
Pyruvate.g.L                        2587    1
Succinate.g.L                       1602    1
Lactate.g.L                         1812    1
Formate.g.L                         2291    1
Acetate.g.L                         2142    1
Intracellular.Mevalonate..uM.        723    1
...

WAIC:  2993.889 .
```

The function output has been truncated for readability (note the ...). `Eff.Sample` is a measure of effective sample size, and `Rhat` is the scale reduction factor on split chains and should be close to 1 at convergence. The output also includes means (`Estimate`), standard deviations (`Est.Error`) and Monte Carlo standard errors (`MC.Error`) if multiple chains are fitted. Due to the large number of metabolomic and proteomic variables, it is not convenient to display all model parameters. As `iCARH.model` returns a `stanfit` object that can be used independently of the package, diagnostic plot functions available in `rstan` can be applied to `fit`. A histogram of `Rhat` values can be plotted by typing `plot(fit$icarh , plotfun="rhat")` (See figure 1). The `traceplot` function displays trace plots of selected posterior distributions of model parameters of the E.coli dataset. By means of example, figure 1 show traceplots of σ_{γ_2}, $\sigma_{\beta_{18}}$, σ, $\phi_4^{controls}$ (`phi[4,1]`) and ϕ_4^{cases} (`phi[4,2]`) for a selected pathway for 1000 iterations.

In order to assess our model fit we carry out similar checks to the previously fitted

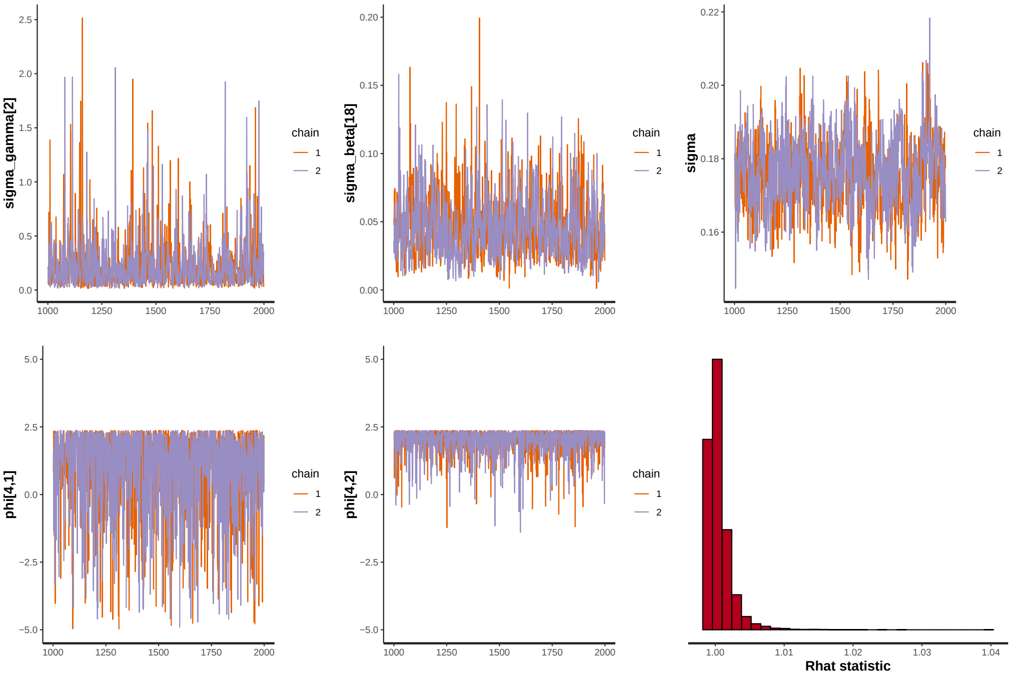

Figure 1: Top panels, bottom right and middle panels show traceplots of σ_{γ_2}, $\sigma_{\beta_{18}}$, σ, $\phi_4^{controls}$ (`phi[4,1]`) and ϕ_4^{cases} (`phi[4,2]`). The bottom right plot shows the `Rhat` statistic for the fitted iCARH model for E.coli dataset indicating that model has converged.

metformin dataset in section 3.2. Figure 2 depicts quantile-quantiles plots of the cases and control groups of the E.coli dataset with a slightly better fit for the cases. This can be explained by the heterogeneity amongst the control strains comprising both the wild type and the engineered unoptimized strains. In addition, we perform posterior predictive checks by computing MADs and comparing our model performance to DPPCA. The texttt below runs normality checks on `fit` and creates quantile-quantile plots and computes MADs using `iCARH`:

```
R> iCARH.checkNormality(fit)
R> mads = iCARH.mad(fit)
```

Histograms in figure 3 show distribution of the logarithm of MADs for DPPCA and iCARH. As observed in the previous analysis, iCARH shows better stability than DPPCA. In fact, as most MADs are smaller than 1 (log(MADs) is less than 0), some MADs reach the unreasonable value of 403 leading to a local maximum around 5. iCARH computed MADs show less variability and are more consistent with log(MADs) ranging from 0.4 to 1.8.

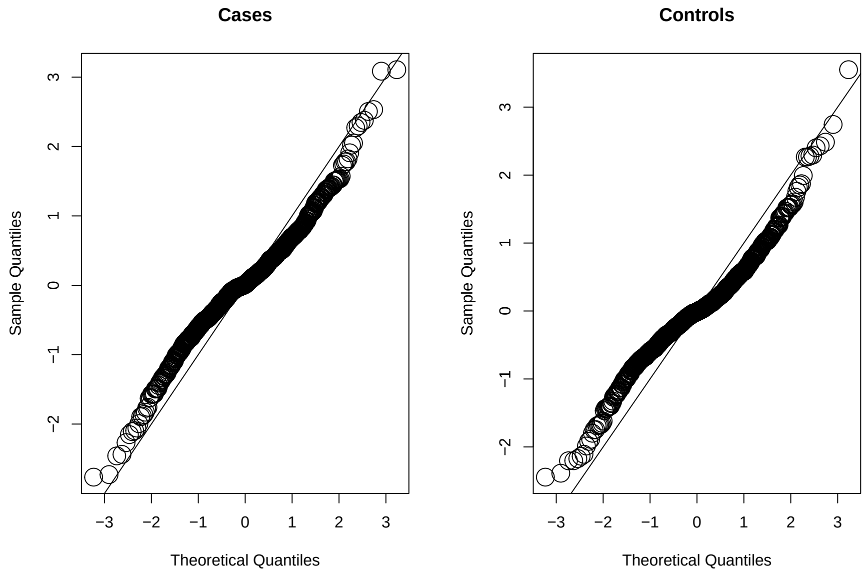

Figure 2: Right and left panels show model fit assessment for controls and cases for E.coli data. Left : quantile-quantile normal plot of $\Psi^{-1}_{\text{cases}}(x_{it} - \mu_{it})$. Right : quantile-quantile normal plot of $\Psi^{-1}_{\text{controls}}(x_{it} - \mu_{it})$.

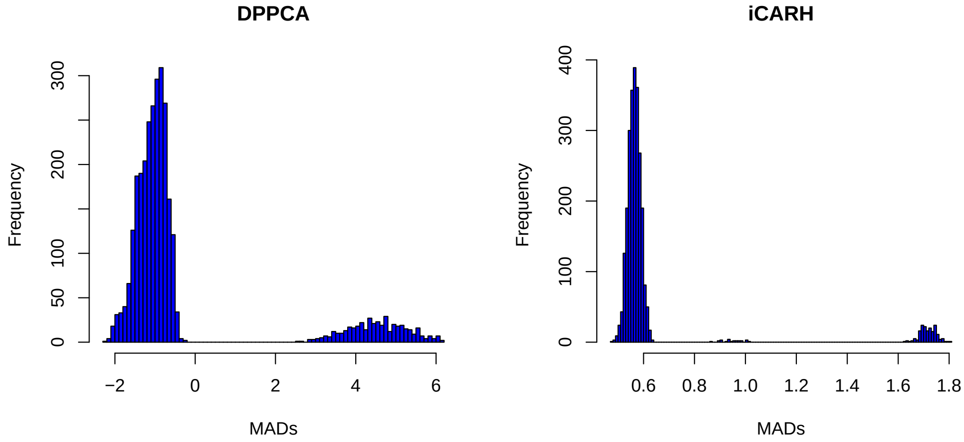

Figure 3: Right and left panels depicts histograms of log(MADs) for iCARH and DPPCA respectively for the E.coli data. The latter shows high variability in the MADs of DPPCA whilst iCARH computed MADs show better stability.

Data results

The output of the `iCARH.model` function is rich and there are different results that can be explored after fitting the iCARH model. We will first show how to display missing

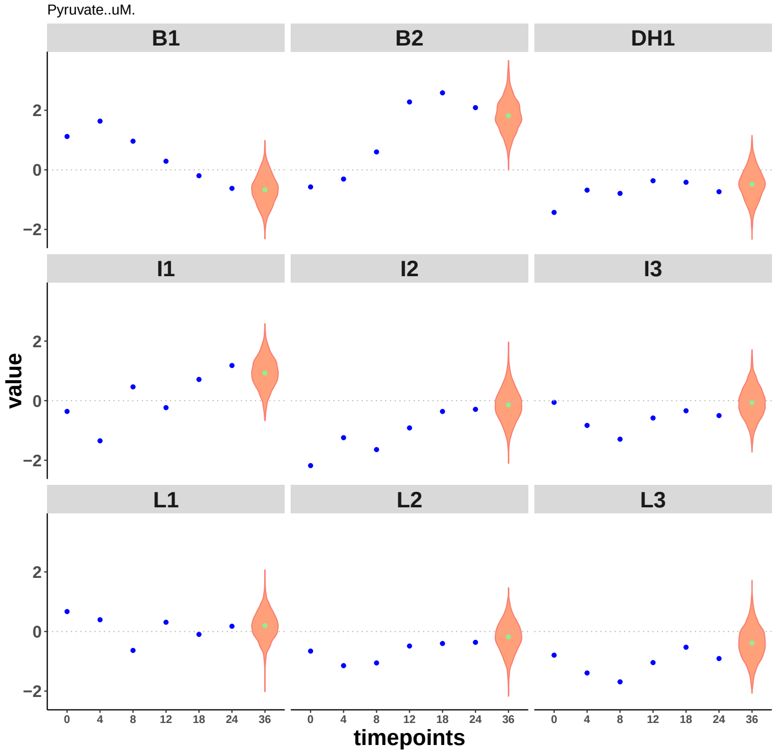

Figure 4: Estimates and posterior distributions of inferred values for missing data points in `Pyruvate..uM` for the E.coli data. Each subplot represents time series for one strain. Blue: Observed data points. Green: Estimated mean values for missing data points. Orange: Posterior distributions of estimates of missing data values.

values imputation before investigating model parameters of interest. Particularly, the function `iCARH.getDataImputation` returns the imputed missing values for both the metabolomics and proteomics data. In addition, `iCARH.plotDataImputation` provides an elegant way to plot imputed values and can take as input particular metabolite or protein indices.
`iCARH.plotDataImputation` produces an output as in figure 4 where each subplot represents time series for one strain. Observed data points are in blue whereas inferred mean values for missing data points are in green. The posterior distribution of inferred values for a missing data point is also computed and displayed as a violin plot in orange.

One of the first effects of interest for applied researchers are longitudinal effects and the treatment effect. Longitudinal effects are captured by θ_m for each metabolite and can be investigated as follows:

```
R> iCARH.plotARCoeff(fit)
```

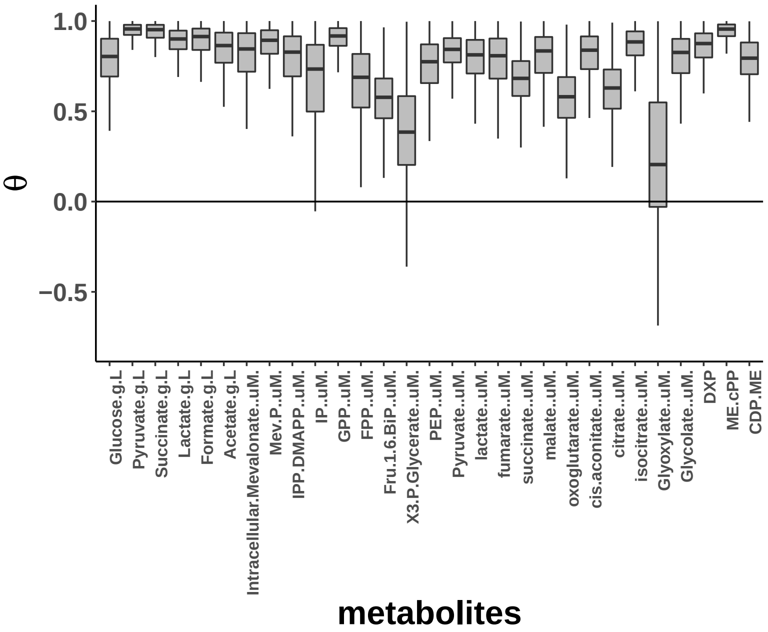

Figure 5: Estimates of effects of time-scale on metabolite profiles are captured by θ_m. Figure depicts boxplots of 95% credible intervals of posterior means of θ_m. The closer the values of θ_m are to 1 the more important time dependence is.

Non significant time dependency is indicated by a low value of θ_m (closer to zero) whereas significance with respect to time scale is indicated by θ_m closer to 1. Namely, in this case glyoxylate does not show significant time dependence. For this dataset, $\boldsymbol{\alpha}_m$ represents an indicator variable for the treatment effect. As the dataset was centered with respect to the control group, a non-zero shift in $\boldsymbol{\alpha}_m$ indicates significant changes in metabolite concentrations as a result of the engineered optimization of the native mevalonate pathway. In `iCARH`, these effects can be directly examined by running

```
R> iCARH.plotTreatmentEffect(fit)
```

which produces the plot in figure 6. Boxplots depict slight changes in metabolite concentrations for the engineered strains. We note that inositol triphosphate (IP) shows the highest shift whereas glyoxylate metabolism related metabolites are not significantly perturbed. In addition, intracellular central carbon metabolites, such as succinate and phosphoenolpyruvate (PEP), and citrate cycle (TCA) metabolites, namely citrate, do not exhibit informative shifts. However, other TCA cycle-related metabolites such as pyruvate (end of glycolysis) and cis-aconitate exhibit higher shifts.

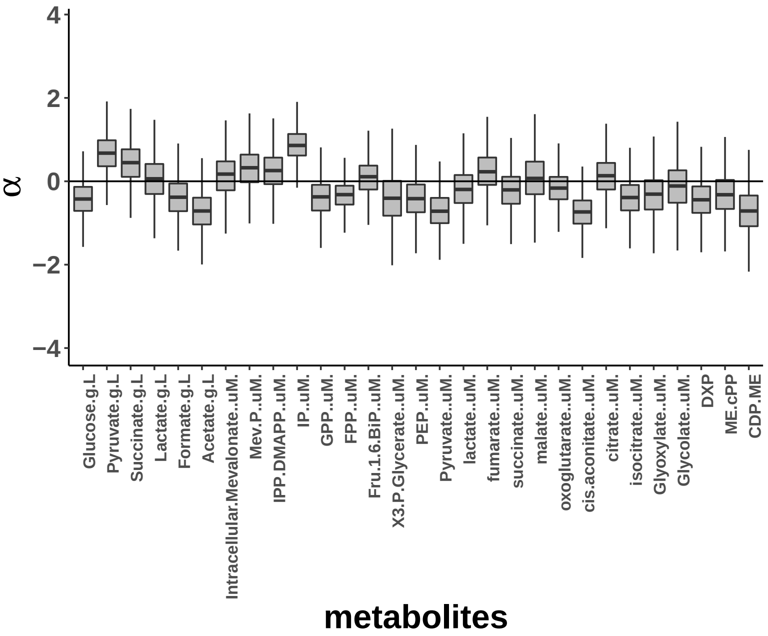

Figure 6: Estimates of effects of optimization strategy on metabolite profiles are captured by $\boldsymbol{\alpha}_m$. Figure depicts boxplots of 95% credible intervals of posterior means of $\boldsymbol{\alpha}_m$.

To elucidate these metabolic perturbations in the context of metabolic pathways, we examine the pathway perturbation plot of the `iCARH.plotPathwayPerturbation` function in figure 7. Some pathways exhibit notable differences between the distribution of the pathway perturbation coefficients $\boldsymbol{\phi}^e$ for the control and cases groups. Most importantly, TCA cycle and pyruvate metabolism show concentrated densities of the corresponding pathway coefficient around the positive eigenvalue which potentially indicates increasing production compared to controls. These findings support results of Brunk et al. (2016) suggesting that TCA cycle reactions and pyruvate pathway are the main drivers of variation.

In order to contextualize our analysis within a biological systems level and get further insights into the underlying mechanisms, the next step of analysis consists in investigating associations between metabolic and proteomic variables. Estimates of effects of proteins on metabolite profiles are captured by $\boldsymbol{\beta}_m$. Overall, the model indicates higher sparsity in the $\boldsymbol{\beta}_m$ coefficients compared to the metformin dataset. The output of the function `iCARH.plotBeta` for some selected proteins is shown in fig 8. Some metabolites present significant changes along with the proteomic profiles. Namely, HMGR protein is negatively associated with pyruvate which is consistent with the pyruvate pathway and positively associated with mevalonate which consistent with the mevalonate pathway. FUMA and NudB proteins exhibit positive association with oxoglutarate. FUMA is an active protein in the TCA cycle and NudB is an active protein in the mevalonate

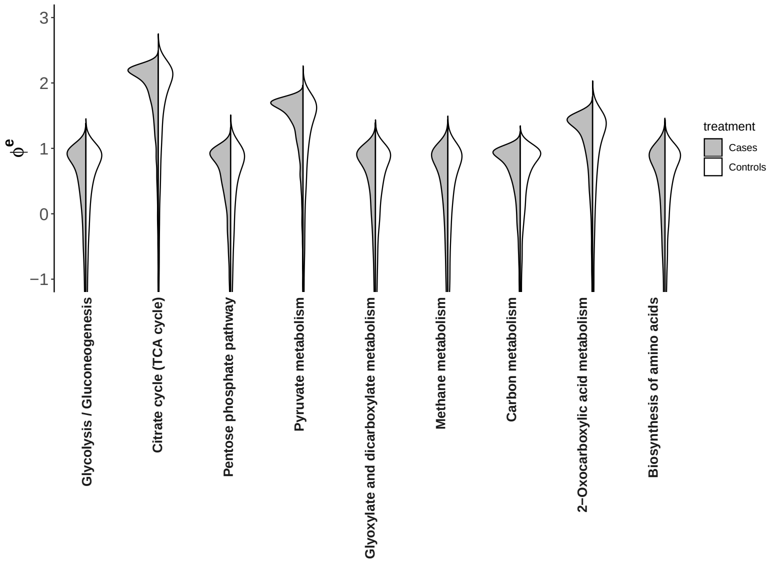

Figure 7: Posterior distributions of $\boldsymbol{\phi}^e$ for each pathway for each treatment group for the E.coli data. Posterior distributions of $\boldsymbol{\phi}^e$ for some pathways are significantly flatter for the controls than the cases which might be indicative of mild pathway alterations.

pathway. The association between these proteins and oxoglutarate are consistent with the perturbation observed in the TCA cycle in 7. To a large extent, our findings are in accordance with the results presented in Brunk et al. (2016) which supports the use of our single model to capture the different sources of variation compared to a multi-step framework assuming independence between these sources.

References

Brunk, E., George, K. W., Alonso-Gutierrez, J., Thompson, M., Baidoo, E., Wang, G., Petzold, C. J., McCloskey, D., Monk, J., Yang, L., et al. (2016). Characterizing strain variation in engineered e. coli using a multi-omics-based workflow. *Cell systems*, 2(5):335–346.

Chong, J., Soufan, O., Li, C., Caraus, I., Li, S., Bourque, G., Wishart, D. S., and Xia, J. (2018). Metaboanalyst 4.0: towards more transparent and integrative metabolomics analysis. *Nucleic acids research*, 46(W1):W486–W494.

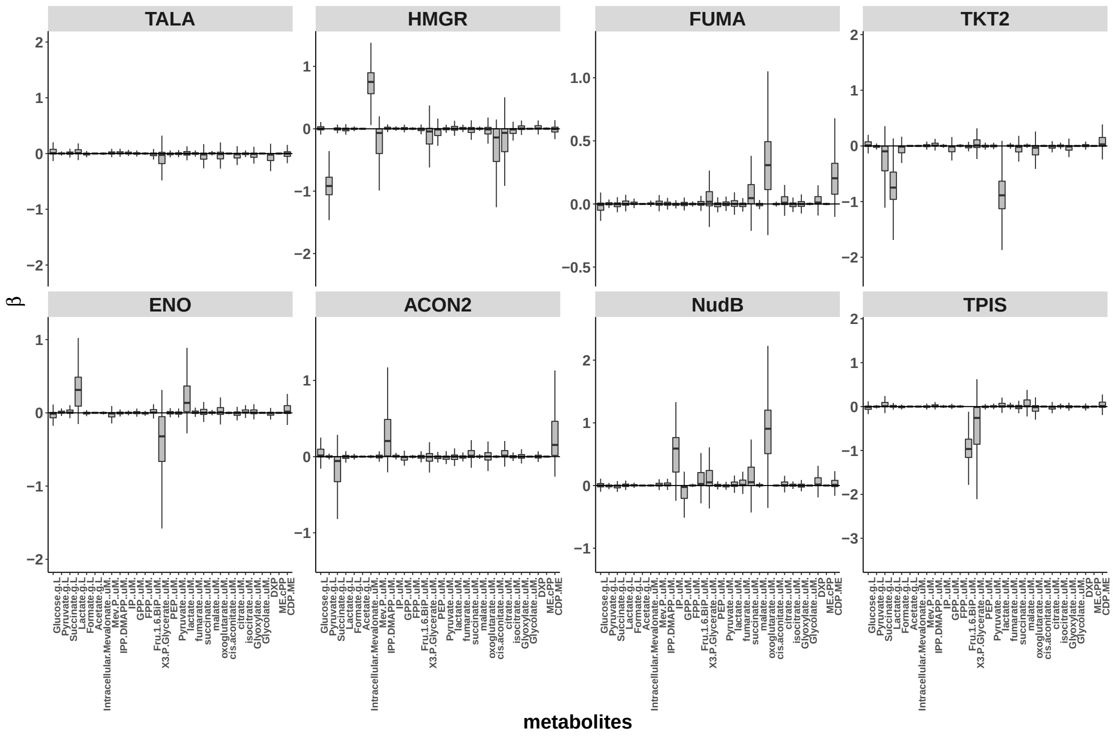

Figure 8: E.coli data: Estimates of effects of proteins on metabolite profiles are captured by $\boldsymbol{\beta}_m$. Figure depicts boxplots of 95% credible intervals of posterior means of $\boldsymbol{\beta}_m$. The sparsity level observed for the E.coli data is higher than the sparsity level observed for the metformin data.
